# Th17/Treg balance is regulated by myeloid‐derived suppressor cells in experimental autoimmune myocarditis

**DOI:** 10.1002/iid3.872

**Published:** 2023-06-14

**Authors:** Xin Xiong, Mengjia Yu, Dinghang Wang, Yange Wang, Longxian Cheng

**Affiliations:** ^1^ Department of Pediatrics, Tongji Hospital, Tongji Medical College Huazhong University of Science and Technology Wuhan China; ^2^ Department of Cardiovascular Surgery, Union Hospital, Tongji Medical College Huazhong University of Science and Technology Wuhan China; ^3^ Department of Emergency, Union Hospital, Tongji Medical College Huazhong University of Science and Technology Wuhan China; ^4^ Department of Cardiology the First Affiliated Hospital of Zhengzhou University Zhengzhou China; ^5^ Laboratory of Cardiovascular Immunology, Institute of Cardiology, Union Hospital, Tongji Medical College Huazhong University of Science and Technology Wuhan China

**Keywords:** autoimmune myocarditis in experiment, balance, inflammation, myeloid‐derived suppressor cells, Th17 cells, Treg

## Abstract

**Objective:**

Autoimmune myocarditis is caused by both innate and adaptive immune responses. Many studies have found that myeloid‐derived suppressor cells (MDSCs) suppress T‐cell responses and reduce immune tolerance, while MDSCs may serve as a key player in inflammatory responses and pathogenesis in variety of autoimmune diseases. However, research into the role of MDSCs in experimental autoimmune myocarditis (EAM) remains lacking.

**Methods and Results:**

We discovered that the expansion of MDSCs in EAM was closely related to the severity of myocardial inflammation. At an early stage of EAM, both adoptive transfer (AT) and selective depletion of MDSCs could inhibit the expression of IL‐17 in CD4^+^ cells and downregulate the Th17/Treg ratio, alleviating excessive inflammation of EAM myocarditis. In another experiment, in addition, MDSCs transferred after selective depletion could increase IL‐17 and Foxp3 expressions in CD4^+^ cells, as well as the Th17/Treg ratio, contributing to the aggravation of myocardial inflammation. MDSCs promoted the Th17 cell induction under Th17‐polarizing conditions in vitro but suppressed Treg expansion.

**Conclusion:**

These findings suggest that MDSCs play a plastic role in sustaining mild inflammation in EAM by shifting Th17/Treg balance.

## Graphical abstract

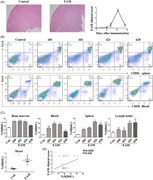

## 1 INTRODUCTION

Myocarditis is distinguished by myocardial inflammation and nonischemic myocytic necrosis as leading cause of sudden cardiac death and dilated cardiomyopathy. It has been established that activated antigen‐specific T lymphocytes play an important role in the pathogenesis of myocarditis,^1^ and a mouse model of experimental autoimmune myocarditis (EAM) has been developed to mimic the immune responses associated with heart‐specific CD4^+^ T cells.^2^, ^3^, ^4^ Th17 cell/Treg axis appears to play a crucial role in the development of autoimmune and inflammatory diseases, including the extent of cardiac inflammation and disease severity.^5^, ^6^

Myeloid‐derived suppressor cells (MDSCs) are immature and progenitor myeloid cells with the ability to suppress immune responses, such as immature macrophages, granulocytes, and dendritic cells, that proliferate in pathological conditions.^7^ MDSCs in mice have a CD11b^+^Gr‐1^+^ phenotype and can be divided into two major subpopulations based on morphology: CD11b^+^Ly6G^‐^Ly6C^high^ monocytic MDSCs (M‐MDSCs) and CD11b^+^Ly6G^+^Ly6C^low^ polymorphonuclear MDSCs (PMN‐MDSCs).^8^ MDSCs alter the expression of surface markers associated with immunosuppressive function, depending on the environment.^9^ In addition to CD11b and Gr‐1, other markers such as CD115 (M‐CSF receptor), CD124 (IL‐4Rα), CD49d (integrin‐α4), CCR2, CXCR2, CD80, CD38, and PD‐L1 (programmed cell death ligand 1) have been used to identify the suppressive activity and trafficking ability of MDSCs8).^8^, ^9^, ^10^ Previous research has concentrated on the immune suppression of MDSCs, primarily targeting T cells. MDSCs not only directly suppress T cells by limiting the availability of several amino acids, promoting apoptosis, inhibiting T‐cell activation, and preventing homing to lymph nodes, but they also have an indirect effect on T cells by inducing Tregs and impairing the ability of antigen‐presenting cells (APCs).^11^ MDSCs have been reported to clear pathogens, provide a niche for pathogens, enhance inflammation, and involved in pathogenesis of various autoimmune diseases.^12^, ^13^, ^14^ MDSCs promoted Th17 cell differentiation in an IL‐1dependent manner and were important pro‐inflammatory factors in experimental autoimmune encephalomyelitis and collagen‐induced arthritis.^15^, ^16^ Shilpak et al. discovered that by secreting cytokines such as TGF, retinoic acid, and arginase‐1 (Arg‐1),^17^ induce TH17 cells from naïve CD4^+^ T cells. Furthermore, tumor‐induced PMN‐MDSCs inhibit TGF‐1‐mediated CD4^+^CD25^+^Foxp3^+^ Treg generation via ROS and indoleamine 2,3‐dioxygenase.^18^ The function of MDSCs in autoimmune diseases appears to be complex and contradictory.

In this study, we used an EAM model to simulate the immune injury of myocarditis and look at how MDSCs function in immune responses. We show that MDSCs play a dual role in EAM by regulating the Th17/Treg balance and maintaining a mild inflammation of myocardium.

## 2 MATERIALS AND METHODS

### 2.1 Animals

Male BALB/c mice (18–20 g, 6–8 weeks old) were purchased from the Hubei Provincial Center for Disease Control and Prevention and housed in pathogen‐free conditions. The Animal Care and Utilization Committee of Huazhong University of Science and Technology approved all animal experiments that followed NIH guidelines (NIH Publication no. 85‐23, revised 1996).

Male BALB/c mice were then randomly assigned to one of the five groups: the Control group, EAM group, AT group, Gem group, and Gem (p)+AT group. On Days 0 and 7, the Control group received a subcutaneous injection with 200 μL of saline. On Days 0 and 7, the EAM group received a subcutaneous injection with 200 μL of the emulsion (250 μg peptide) of the murine MyHC‐peptide synthesized according to the MyHC‐614–629 sequence (acetyl‐SLKLMATLFSTYAS), dissolved in saline (2.5 mg/mL), and emulsified it 1:1 in complete Freund's adjuvant. AT group: intravenous injection of sorted MDSCs (5 × 10^6^) into EAM mice on Days 0 and 7. Gem group: intraperitoneal administered intraperitoneally every three beginning on Day 7, the second immunization with MyHC‐peptide in EAM mice. AT of MDSCs (5 × 10^6^) on Day 14 after a week of gemcitabine treatment (Days 7, 10, and 13) in EAM mice.

### 2.2 EAM induction and evaluation

We dissolved in saline (2.5 mg/mL) the murine MyHC‐peptide synthesized according to the MyHC‐α_614–629_ sequence (acetyl‐SLKLMATLFSTYAS), then emulsified it 1:1 in complete Freund's adjuvant (Sigma‐Aldrich).^2^ EAM mice model was established by subcutaneous injection with 200 μL of the emulsion (250 μg peptide) on Days 0 and 7; accordingly, the Control group received saline as a vehicle at the same time points. On Day 21, we assessed and graded the severity of EAM based on the infiltration of inflammation cells in myocardium: 0, no inflammation; 1, <25% of the heart section affected by inflammation; 2, 25%–50% of the heart section involved in inflammation; 3, 50%–75% of the heart section involved in inflammation; and 4, >75% of the heart section involved in inflammation.^19^ The ratio of heart weight to body weight (HW/BW) was measured to indirectly evaluate myocarditis severity.

### 2.3 Hematoxylin and eosin stain

On Day 21, mice were killed for histopathological examination. We produced paraffin slices, fixed the heart tissues in 10% buffered formalin for 24 h, and stained them with hematoxylin and eosin (H&E; Solarbio). Image‐Pro® Plus 6.0 was used to analyze the inflammation and fibrosis in the microscope at a magnification of 200 to detect myocarditis in mice.

### 2.4 Flow cytometry

We labeled cells with antibodies against markers: anti‐mouse CD11b‐PE (clone: M1/70; BioLegend), anti‐mouse Gr‐1‐FITC (clone: RB6‐8C5; BioLegend), anti‐mouse Ly6G‐FITC (clone: 1A8‐Ly6g; eBioscience), anti‐mouse Ly6C‐APC (clone: HK1.4; eBioscience), anti‐mouse CD4‐FITC (clone: GK1.5; BD Biosciences), anti‐mouse CD4‐APC (clone: GK1.5; eBioscience), anti‐mouse IL‐17A‐PE (clone: TC11‐18H10; BD Biosciences), anti‐mouse Foxp3‐PE (clone: FJK‐16s; eBioscience), anti‐mouse CD124‐PE/Cy7 (IL‐4Rα, clone: I015F8; BioLegend), anti‐mouse CD119‐PE (IFN‐γR1, clone: 2E2; eBioscience), anti‐mouse iNOS‐APC (clone: CXNFT; eBioscience), anti‐mouse CCR2‐APC (clone: 475301; R&D Systems), and anti‐mouse CD197‐PE/Cy7 (CCR7, clone: 4B12; eBioscience). MDSCs and T lymphocytes were surface stained after 30 min at 4°C incubation with relevant antibodies shielded from light. CD4^+^ T cells were stained with anti‐IL‐17A after being stimulated with the Cell Stimulation Cocktail (eBioscience) for 4–6 h to measure intracellular cytokine production. Following incubation in 1× Fixation and Permeabilization Solution, CD4^+^ T was stained with Foxp3 antibody for Treg detection. Data were acquired on a BD FACSCalibur™ flow cytometer (BD Biosciences) and analyzed with FlowJo version 7.6.1 (Treestar).

### 2.5 Isolation of cells and preparation of samples

Grinding the organs through 200 μm mesh sieves yielded single‐cell suspensions from spleens and inguinal lymph nodes. The excised hearts were digested in a water bath at 37°C with 0.1% collagenase B solution (Roche Diagnostics), and then passed through 70 μm cell strainers.^20^ Mononuclear cells and granulocytes were isolated and collected from peripheral blood, bone marrow, spleen, and heart using Lymphocyte Separation Medium (1.0770–1.0800 g/mL; MP Biomedicals) or Histopaque®‐1119 (1.1190 g/mL; Sigma‐Aldrich) and used in subsequent experiments.

### 2.6 MDSC depletion in vivo

To assess the involvement of MDSCs in vivo, we gave gemcitabine (100 µg/g; MedChem Express) intraperitoneally to EAM mice at 3‐day intervals from Day 7 after the second immunization.^21^ The protocol is detailed in the figures and legend.

### 2.7 Cell purification and AT

Splenic MDSCs from EAM mice were purified on Day 21 using Ly6G^+^Gr‐1^+^ magnetic beads (Miltenyi Biotec) per the manufacturers’ instructions.^22^ Wright–Giemsa was used to fix and stain the cells. CD11b^+^ cells made up. approximately 98% of the Ly6G^+^Gr‐1^+^ cells were CD11b^+^. In adoptive transfer (AT) experiments, enriched MDSCs (5 × 10^6^ cells) were intravenously transferred into his Day 7. Another group of mice received his MDSC transplant (5 × 10^6^ cells) after gemcitabine treatment.^15^

### 2.8 Proliferation of T cells

Lymphocytes from healthy mice spleens were labeled with 2.5 μM 5(6)‐carboxyfluorescein diacetate succinimidyl ester (CFSE; eBioscience) and used as responder T lymphocytes. We investigated MDSC‐mediated immune suppression by coculturing with CFSE‐labeled T cells activated with plate‐bound anti‐mouse CD3 (5 μg/mL; eBioscience) and soluble anti‐mouse CD28 (5 μg/mL; eBioscience) for 72–96 h at different cell ratios (MDSCs/T cells ratios: 0:1, 1:8, 1:4, 1:2, 1:1, and 2:1) in complete RP (Gibco). We assessed the suppression of T‐cell proliferation using CFSE fluorescence intensity, and the development of T cells using flow cytometry to detect Th17 cells and Tregs. In some experiments, we used the arginase inhibitor Nω‐hydroxy‐nor‐l‐arginine (nor‐NOHA; Calbiochem) or the iNOS inhibitor NG‐monomethyl‐l‐arginine (L‐NMMA; Sigma‐Aldrich) at a concentration of 500 µM.^23^

### 2.9 Th17 and Treg cell generation

Splenic CD4^+^ cells were isolated from splenocytes and cocultured with Ly6G^+^Gr‐1^+^ MDSCs in a 4:1 ratio using CD4+ magnetic beads (Miltenyi Biotec). For Th17 cell induction, T cells stimulated with 5 μg/mL anti‐CD3 and 5 μg/mL anti‐CD28 were incubated in duplicate in the presence of 1% penicillin/streptomycin, recombinant mouse TGF‐β1 (2 ng/mL; R&D Systems), recombinant mouse IL‐23 (10 ng/mL; R&D Systems), recombinant mouse IL‐2 (2 ng/mL, PeproTech), and recombinant mouse IL‐6 (20 ng/mL, PeproTech) at 37°C in complete RPMI 1640 medium (Hyclone) containing 5% fetal bovine serum (Gibco) for 7 days. Similarly, we looked at MDSCs affected Treg differentiation in Treg‐skewing conditions with 2.5 ng/mL TGF‐β after 5 days of anti‐CD3 and anti‐CD28 simulation. Using low cytometer, we determined the frequencies of Th17 cells and Tregs.

### 2.10 RT‐qPCR

Total RNA was extracted from tissues and cells using TRIzol™ lysis buffer (Takara Bio), then treated with a Reverse Transcriptase kit (Takara) to synthesize cDNA, according to the manufacturer's protocols. Gene expression was quantified using an SYBR® Green PCR Kit (Takara) and an ABI Prism 7900 Sequence Detection System (Applied Biosystems). The sequences of the primers are shown in Table 1. The relative levels of gene expression were normalized to the housekeeping gene GAPDH with the standard $2^{‐∆∆C_{t}}$ calculation.

**Table 1:** The primer sequences used for real‐time PCR.

Gene	Primer sequences (5′‐3′)
GAPDH	Forward: AGGTCGGTGTGAACGGATTTG
	Reverse: GGGGTCGTTGATGGCAACA
TNF‐α	Forward: CAGGCGGTGCCTATGTCTC
	Reverse: CGATCACCCCGAAGTTCAGTAG
IL‐1β	Forward: CTGGTACATCAGCACCTCAC
	Reverse: AGAAACAGTCCAGCCCATAC
IL‐10	Forward: GCTCTTACTGACTGGCATGAG
	Reverse: CGCAGCTCTAGGAGCATGTG
TGF‐β	Forward: CTCCCGTGGCTTCTAGTGC
	Reverse: GCCTTAGTTTGGACAGGATCTG
Foxp3	Forward: GGCAGAGGACACTCAATGAAAT
	Reverse: TCTCCACTCGCACAAAGCAC

### 2.11 Cytokine detection

The supernatants were collected from the T‐cell proliferation assays and centrifuged at 3000*g* for 15 min. The concentrations of IFN‐γ, IL‐1β, IL‐6, IL‐10, and TGF‐β were measured with enzyme‐linked immunosorbent assay kits (Hangzhou MultiSciences Biotech) in accordance with the manufacturer's instructions.

### 2.12 Echocardiography

Echocardiography was performed using a Sonos 5500 ultrasound machine (Hewlett‐Packard Co.) and a 15 MHz linear transducer (Agilent Technologies). Animals were anesthetized with isoflurane on Day 21 after induction of EAM. After a short‐axis 2‐D image of the LV was obtained at the level of the papillary muscles, 2D guided M‐mode images were acquired at a sweep speed of 100 mm/s and stored digitally. The LV anterior wall at diastole (LVAWd) and systole (LVAWs) and the LV inner dimensions at diastole (LVIDd) were measured digitally on the M‐mode trace using the leading‐edge technique.

### 2.13 Statistical analysis

The mean ± standard error of mean (SEM) was used to present the results. GraphPad Prism 5.0 was used for visualization and statistical analysis, and comparison between the two groups was analyzed using Student's *t* test. The data were analyzed by one‐way ANOVA followed by Bonferroni's multiple comparison test between different groups. Potential linear correlations between the variables were evaluated with the Spearman correlation analysis. Differences with *p* < .05 were considered statistically significant.

## 3 RESULTS

### 3.1 MDSCs dynamics in EAM

**Figure 1:**
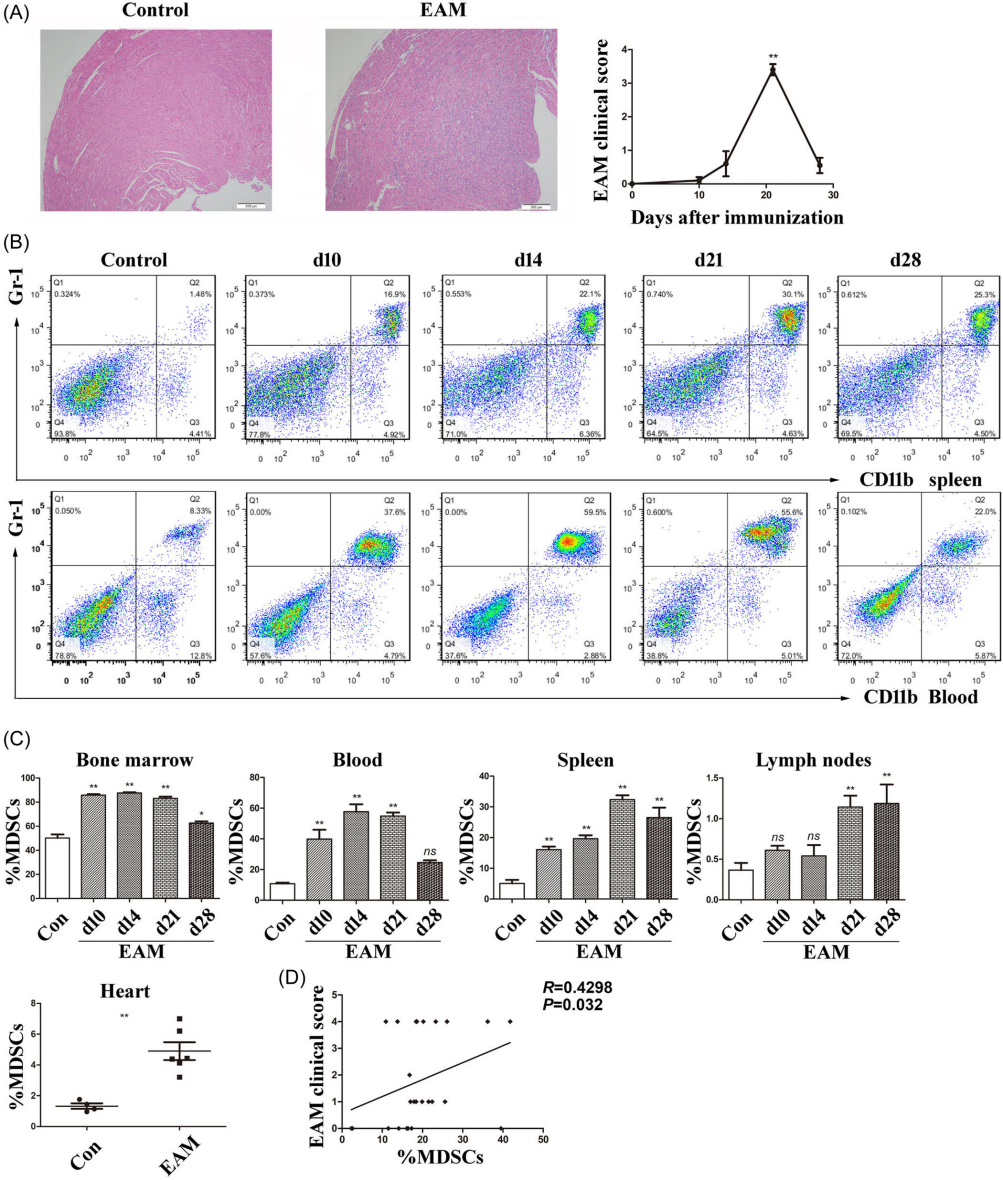
The proportion of MDSCs in Control and EAM mice. (A) On Day 21, H&E‐stained heart sections from Control and EAM mice. The EAM clinical scores were measured at each time point (Days 0, 10, 14, 21, and 28) following immunization of the EAM mice with MyHC‐α (*n* = 5 mice/group). (B) Representative flow cytometric images of CD11b^+^Gr‐1^+^ MDSCs in the spleens or blood of Control and EAM mice at different stages of EAM development (d10, d14, d21, and d28). (C) The dynamic percentages of CD11b^+^Gr‐1^+^ MDSCs from Control and EAM mice tissues (*n* = 4–11 mice/group). The percentages of CD11b+Gr‐1+ MDSCs in immunocytes from the heart of Control and EAM mice on Day 21 (*n* = 4–6 mice/group). (D) Spearman correlation analysis of MDSC percentages and EAM clinical scores at different stages of EAM (*n* = 25 mice). Values are means ± SEM. Three or more independent experiments have been conducted. ^ns^ *p* > .05, *p < .05 and **p < .01 versus Control group. EAM, experimental autoimmune myocarditis; H&E, hematoxylin and eosin; MDSC, myeloid‐derived suppressor cell.

To clarify the function of MDSCs in EAM, we constructed a mouse model of EAM.^24^ During the course of EAM, we demonstrated that myocardial inflammation appeared on his 10th day, progressed on his 14th day, peaked on his 21st day, and then declined (Figure 1A). Echocardiography revealed that EAM mice had an increased anterior wall thickness (LVAW) with suppressed cardiac output, left ventricular ejection fraction (LVEF), and LV fractional shortening (FS) when compared with those measured in Control mice (Supporting Information: Figure S1A,B). We observed impaired systolic function and mild ventricular remodeling in myocarditis progression.

Interestingly, the percentage of splenic CD11b^+^Gr‐1^+^ MDSCs changed dynamically following the inflammatory process, whereas blood MDSCs peaked approximately 7 days before splenic MDSCs (Figure 1B). We have studied the kinetics of MDSCs frequencies in various tissues. These results suggest that the majority of MDSCs were released from the bone marrow into the blood and then transported to heart and secondary lymphoid tissues (Figure 1C). To investigate the relationship between the frequency of splenic MDSCs frequencies and the severity of myocarditis, a correlation analysis was performed, suggesting that the percentage of splenic CD11b^+^Gr‐1^+^ MDSCs was positive in the clinical EAM score (Figure 1D, *R* = .4298, *p* = .032).

### 3.2 Characterization of MDSCs in EAM mice

**Figure 2:**
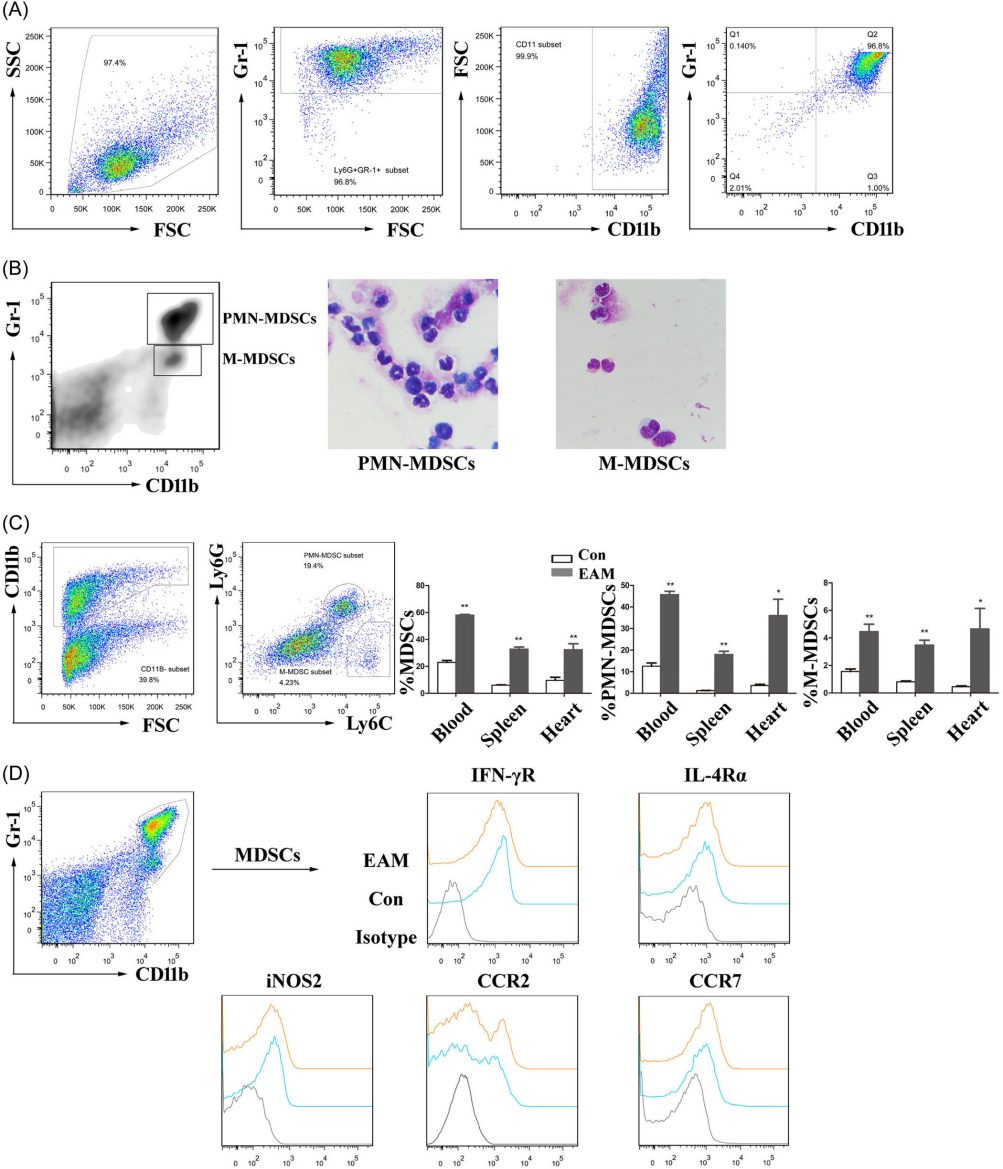
MDSC characterization in EAM mice. (A) MDSC purity as determined by Ly6G+Gr‐1+ magnetic beads. (B) Wright–Giemsa staining of PMN‐MDSCs (Ly6G+ cells) and M‐MDSCs (Gr‐1+ cells) isolated from Control and EAM mice spleens. (C) Representative flow cytometric images of PMN‐MDSCs (CD11b^+^Ly6G^high^Ly6C^low^ cells) and M‐MDSCs (CD11b^+^Ly6G^low^Ly6C^high^ cells) in the EAM mouse heart. On Day 21, statistical analysis revealed the percentages of MDSC phenotypes in the blood, spleens, and hearts of Control and EAM mice (*n* = 4–5 mice/group). (D) The expression of functional and chemotactic markers (IFN‐γR, IL‐4Rα, iNOS2, CCR2, and CCR7) on splenic CD11b^+^Gr‐1^+^ MDSCs from Control and EAM mice. At least three independent experiments have been carried out. *p < .05 and **p < .01 versus the Control group. EAM, experimental autoimmune myocarditis; MDSC, myeloid‐derived suppressor cell; PMN, polymorphonuclear.

We next characterized MDSCs from EAM mice by flow cytometry and Wright–Giemsa staining. MDSC was greater than 95% as sorted by Ly6G^+^Gr‐1^+^ magnetic beads, suggesting that PMN‐MDSC shows overwhelming predominance in EAM mice (Figure 2A). To determine additional markers for MDSCs on Day 21 of EAM, we first isolated two major subsets of MDSCs obtained from the spleens of EAM mice, M‐MDSCs (Gr‐1^+^ cells) and PMN‐MDSCs (Ly6G^+^ cells) were examined. Wright–Giemsa staining showed that M‐MDSCs were mononuclear, whereas PMN‐MDSCs displayed her PMN granulocyte morphology (Figure 2B). The percentages of the CD11b^+^ cells, PMN‐MDSCs, and M‐MDSCs in the blood, spleen, and heart of EAM mice were more significant than those of Control mice (Figure 2C). We also compared the expression of markers associated with cell function and chemotaxis (Figure 2D). Enriched splenic MDSCs in EAM mice increased the absolute number of functional and chemotactic MDSCs, whereas the expression levels of IFN‐γR, IL‐4Rα, iNOS2, CCR2, and CCR7 in MDSCs of EAM mice have no significant differences compared with Control mice.

### 3.3 AT and MDSC depletion slowed EAM progression

**Figure 3:**
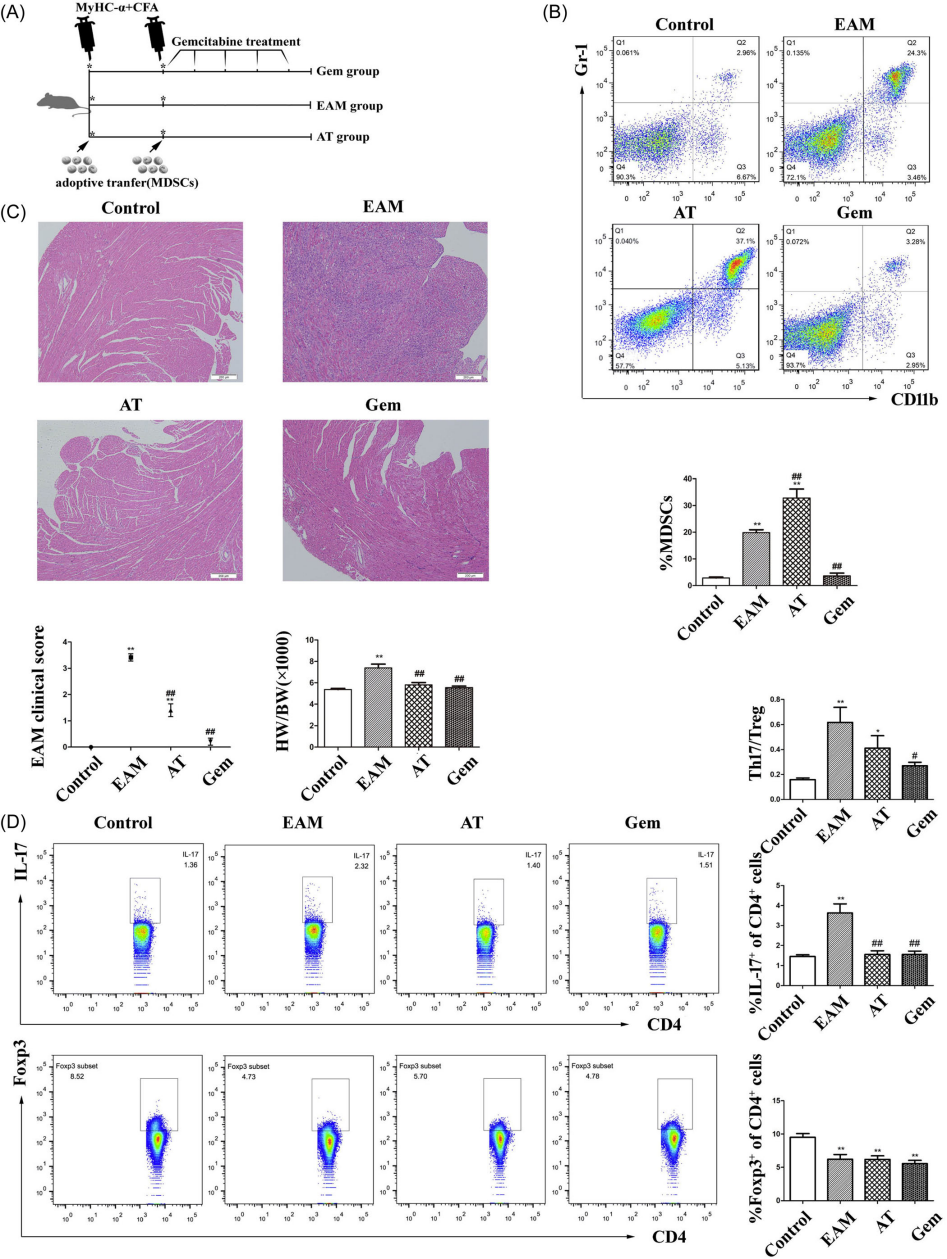
Adoptive transfer (AT) and MDSC depletion improved EAM progression. (A) A flow chart of mice in the AT and Gem groups. On Days 0 and 7, AT was carried out via intravenous injection of sorted MDSCs (5 × 10^6^ cells). We depleted MDSCs by administering gemcitabine intraperitoneally every 3 days beginning on Day 7 after the second immunization. (B) Flow cytometry was used to determine the proportion of CD11b^+^Gr‐1^+^MDSC in mouse spleen (*n* = 5 mice/group). (C) H&E‐stained cardiac tissue damage in mice was assessed, and the EAM clinical score and the HW/BW ratio in mice were statistically analyzed (*n* = 4–15 mice/group). (D) Flow cytometry was used to analyze the ratio of Th17 cells and Tregs in the mice spleens of each group (*n* = 4–7 mice/group). Three or more independent experiments have been conducted. *p < .05 and **p < .01 versus Control group, ^#^ *p* < .05 and ^##^ *p* < .01 versus EAM group. EAM, experimental autoimmune myocarditis; H&E, hematoxylin and eosin; MDSC, myeloid‐derived suppressor cell.

MDSCs have the ability to suppress immune responses and polarize immunity against anti‐inflammatory conditions. To investigate the potential effects of MDSCs in the progression of EAM, we could increase MDSCs via AT or deplete MDSCs by gemcitabine treatment (Gem) to gain control of MDSCs (Figure 3A). The results showed that the percentage of MDSCs changed significantly in AT and Gem group mice were compared with the EAM group (Figure 3B). As illustrated in Figure 3C, H&E staining revealed that AT and gemcitabine‐treated mice had less inflammatory infiltration than EAM mice, which was consistent with the lower EAM clinical scores and HW/BW ratios. The expression of anti‐inflammatory cytokines (IL‐10 and TGF‐β) was lower in AT or gemcitabine‐treated mice than in EAM mice, whereas the level of the pro‐inflammatory cytokine IL‐1β not statistically different (Supporting Information: Figure S2A). The cytokine profiles showed an anti‐inflammatory inclination of MDSCs to prevent and cure the immune injury. The transfer of EAM MDSCs in early stage of EAM could alleviate the inflammation and myocardial injury as expected. Surprisingly, impeding MDSC release from bone marrow with gemcitabine had the same, if not better, protective effect in EAM progression. These findings, at the very least, suggested a contentious role for MDSCs in the early stage of EAM.

To assess the role of MDSCs in immune responses, we looked at the ratio of Th17 to Treg cells in splenic CD4^+^ cells. As shown in Figure 3D, the expression of IL‐17 in CD4^+^ cells of EAM increased, whereas the expression of IL‐17 CD4^+^ cells of AT or gemcitabine‐treated mice decreased compared to that of EAM mice. The expression of Foxp3 in CD4^+^ cells was significantly reduced in EAM mice, as expected, but Treg suppression remained after the transfer of MDSCs, which was inconsistent with previous findings of MDSCs inducing Treg expansion in vivo.^25^ On the contrary, the mRNA expression of Foxp3 in spleen was decreased in AT mice, although the reasons are not clear (Supporting Information: Figure S3). Furthermore, the Th17/Treg ratios in both AT and gemcitabine‐treated mice were lower than in EAM mice, confirming that the alteration of MDSCs in early stages of EAM could lead to a dampening immune response, consistent with the reduced inflammation in myocarditis.

### 3.4 MDSC transfer played a minor role in the pathogenesis of the late stage of EAM

**Figure 4:**
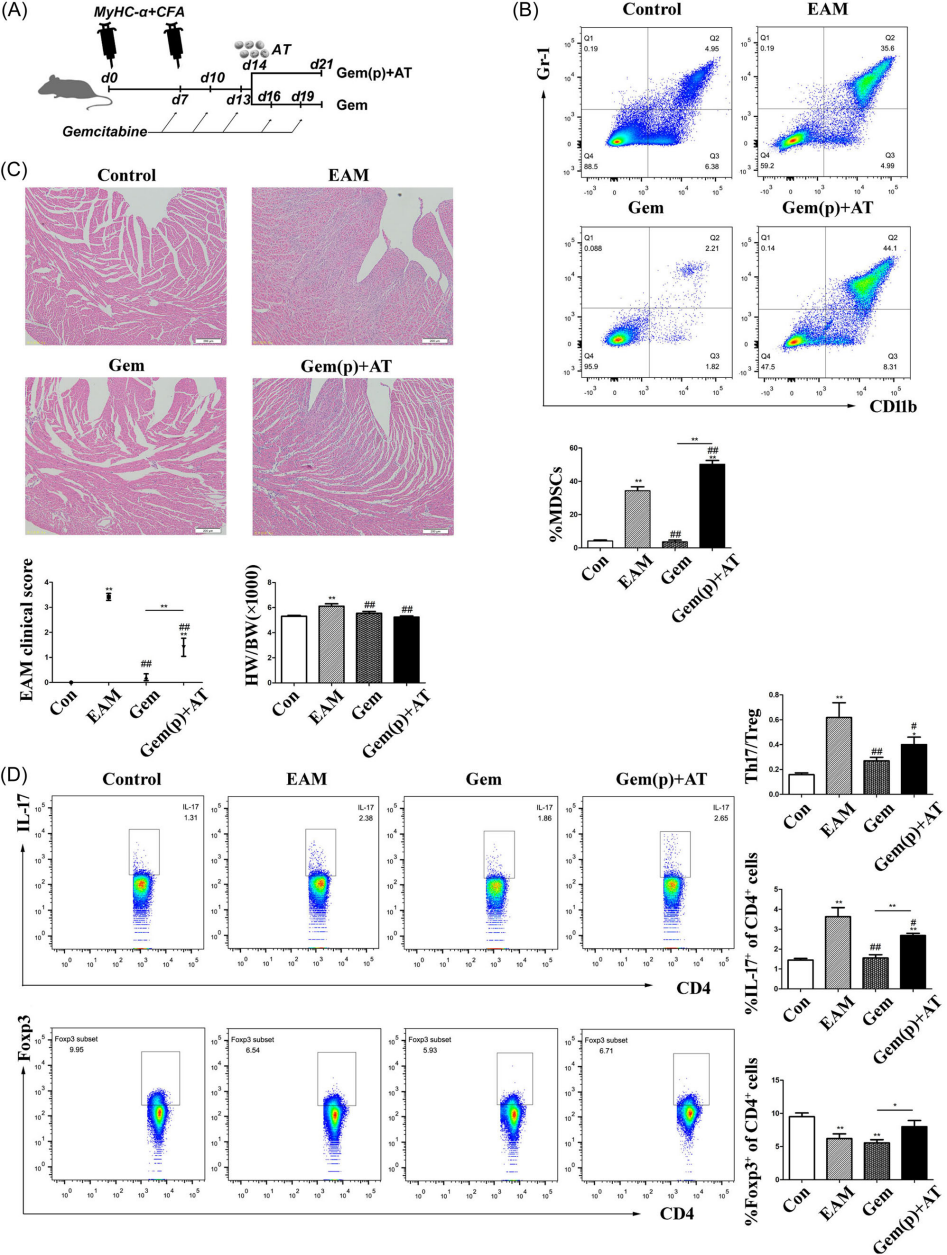
In gemcitabine‐treated EAM mice, MDSC transfer played a minor role in the pathogenesis of the late stage of EAM. (A) The flow chart of mice in the Gem and Gem(p)+adoptive transfer (AT) treated groups. After a week of gemcitabine treatment, adoptive transfer of MDSCs (5 × 10^6^ cells) from EAM mice was performed on Day 14 to observe the accumulation of peripheral MDSCs in later stage. (B) Flow cytometry was used to determine the percentages of CD11b^+^Gr‐1^+^ MDSCs mouse spleen (*n* = 5 mice/group). (C) H&E‐stained cardiac tissue damage in mice was assessed, and the EAM clinical score and the HW/BW ratio in mice were statistically analyzed (*n* = 6–7 mice/group). (D) The ratio of Th17 cells and Tregs in CD4+ cells in the spleens of each group was analyzed by flow cytometer (*n* = 4–8 mice/group). Three or more independent experiments have been conducted. *p < .05 and **p < .01 versus Con group, ^#^ *p* < .05 and ^##^ *p* < .01 versus EAM group. EAM, experimental autoimmune myocarditis; H&E, hematoxylin and eosin; MDSC, myeloid‐derived suppressor cell.

Given the protective effect of MDSCs depletion via gemcitabine treatment in EAM mice, we were skeptical of MDSCs' suppressive function under any circumstances. Some studies have described the various microenvironments that should be responsible for the plasticity of MDSC function during disease progression.^26^, ^27^ To that end, we transferred MDSCs after a period of gemcitabine treatment (Gem (p)+AT mice) (Figure 4A). Because gemcitabine treatment has been shown to inhibit MDSC accumulation in the spleen,^21^ we hypothesized and observed the transfer of MDSCs and the interruption of gemcitabine treatment both promoted the accumulation of peripheral MDSCs in the later stages of EAM (Figure 4B). Unexpectedly, there was a mild increase in clinical myocarditis scores in Gem (p)+AT mice when compared to Control mice and gemcitabine‐treated mice, with no significant difference in the HW/BW ratio (Figure 4C). The mRNA expression of IL‐10 was higher in Gem (pause)+AT mice than in Gem(pause) mice (Supporting Information: Figure S2B). Among abovementioned cytokines, IL‐10 always kept consistent with the inflammation state actively.

Furthermore, after MDSC transfer, both IL‐17 and Foxp3 expression in CD4^+^ cells increased, while the Th17/Treg ratio increased in Gem(p)+AT mice (Figure 4D). The proclivity of MDSCs for Th17 induction suggested that MDSCs could cause a Th17/Treg imbalance and promote myocardial inflammation, triggering later stages of EAM.

### 3.5 In vitro regulation of MDSCs on Th17 cells and Tregs induction

**Figure 5:**
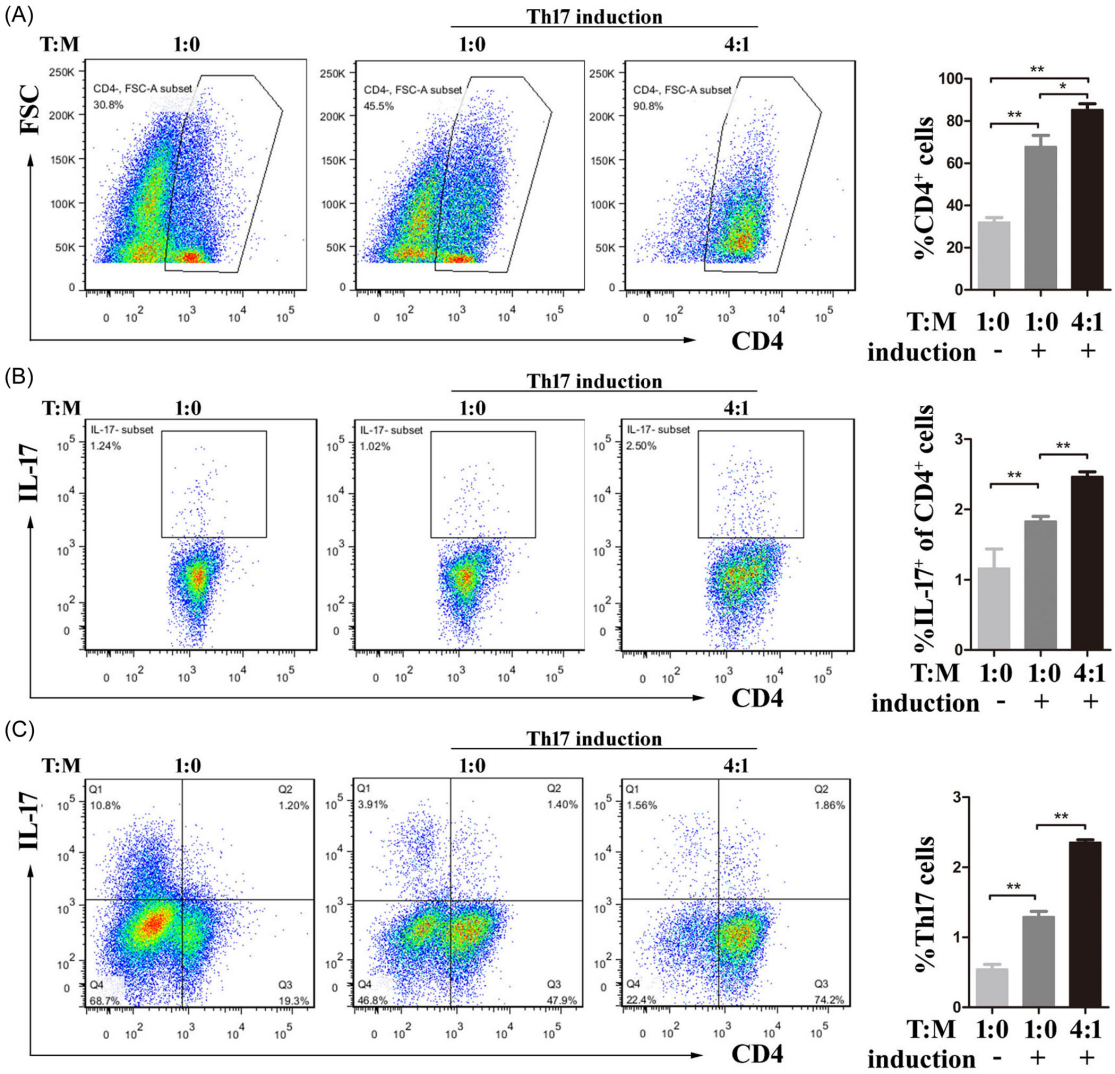
MDSCs induce the differentiation of Th17 cells under Th17‐polarizing conditions in vitro. For 96 h, sorted MDSCs were cocultured with CD4^+^ T cells from mice in the Control group. (A) Flow cytometry was used to determine the percentages of CD4^+^ cells. (B, C) Co‐cultures with MDSCs increased the percentages of IL‐17^+^ cells in CD4^+^ cells (B) and CD4^+^IL‐17^+^ cells (C). * *p* < .05, ** *p* < .01. MDSC, myeloid‐derived suppressor cell.

**Figure 6:**
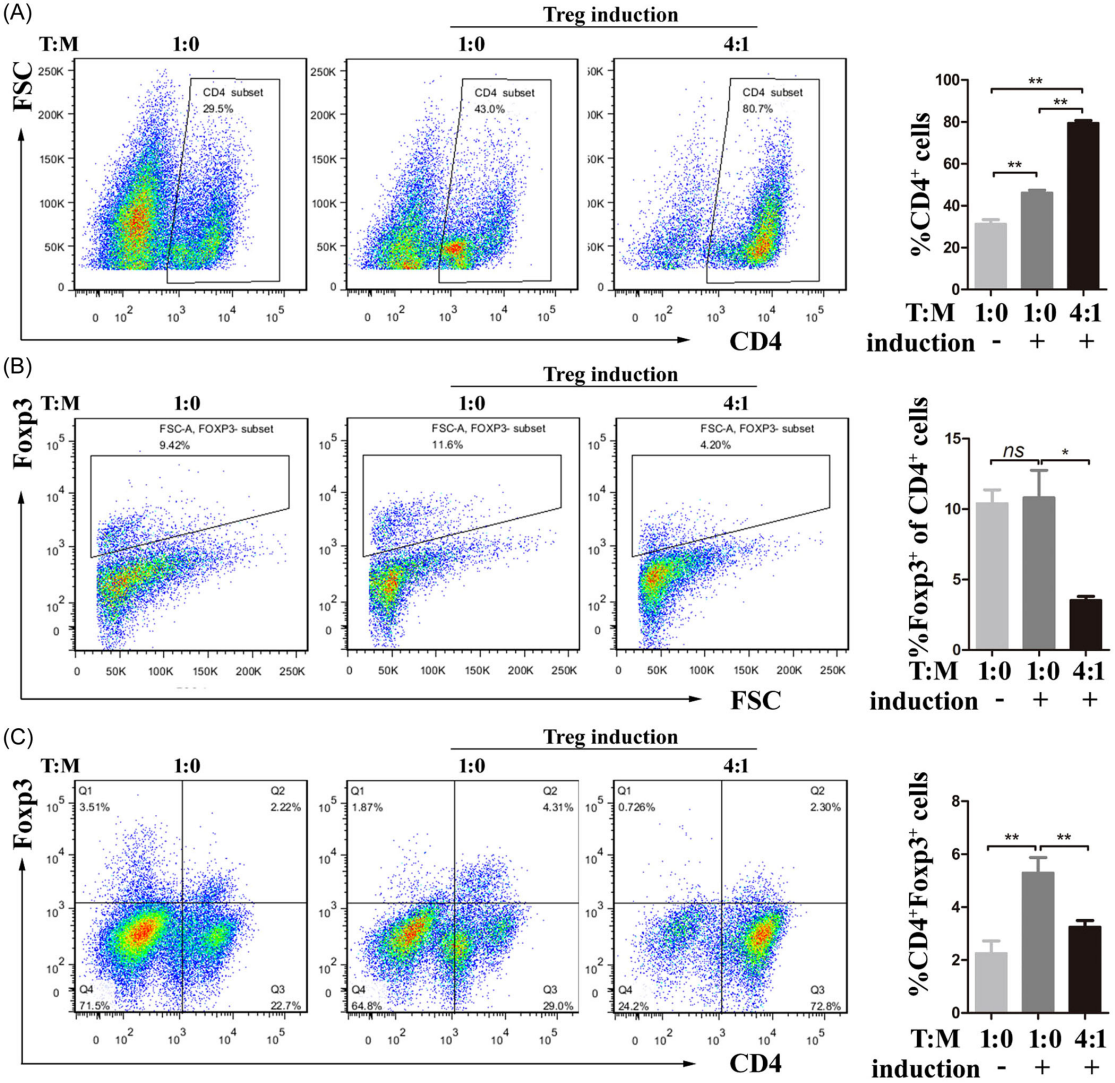
MDSCs inhibit Treg differentiation in vitro under Treg‐polarizing conditions. Sorted MDSCs were cocultured for 96 h with CD4^+^ T cells from Control mice inTreg‐polarizing conditions. (A–C) Co‐cultures with MDSCs, the percentages of CD4^+^ cells (A) were increased, whereas the percentages of Foxp3^+^ cells in CD4^+^ cells (B) and CD4^+^Foxp3^+^ cells (C) were both decreased. At less three or more independent experiments have been carried out. *p < .05, **p < .01 and *ns* denotes nonsignificant. MDSC, myeloid‐derived suppressor cell.

In myocarditis, CD4^+^ T cells are known to be essential effector cells; on the other hand, Th17 cells increase pro‐inflammatory factor release and promote disease progression. An imbalance between Th17 and Treg cells is linked to the onset of inflammation and the development of myocarditis.^6^ To determine the impact MDSCs on the Th17 cell/Treg axis, the development of stimulated T cells cocultured with MDSCs at a 4:1 ratio was first observed. In addition to MDSCs suppressing T‐cell proliferation, the expression levels of IL‐17 and Foxp3 in CD4^+^ T cells were enhanced, along with the obvious production of IFN‐γ, IL‐1β, IL‐6, and IL‐10 (Supporting Information: Figure S4A–C). We then cocultured splenic CD4^+^ T cells with MDSCs in Th17‐ or Treg‐skewing conditions and CD4^+^ T‐cell expansion (Figures 5A and 6A). In Th17‐skewing conditions, the ratio of Th17 cells in CD4^+^ T cells was significantly increased with the assistance of MDSCs, indicating the induction of MDSCs on Th17 cells (Figure 5B,C). However, in Treg‐skewing conditions, the presence of MDSCs significantly inhibited Foxp3 expression (Figure 6B,C). These findings suggested that MDSCs may have pro‐inflammatory functions in certain circumstances.

## 4 DISCUSSION

MDSCs have been extensively studied in cancer research, and their primary function has been identified as suppressing T‐cell responses.^7^, ^8^ Our findings revealed that MDSCs in EAM displayed variable immune characteristics depending on the environment. MDSCs, in particular, have the potential to correct the imbalance between Th17 cells and Tregs by impairing T‐cell suppressive capacity or inducing T‐cell differentiation, thereby sustaining a low‐grade inflammation.

In general, MDSCs are only detectable in physiological conditions, whereas MDSCs play a role in immune response regulation in a variety of pathological situations, such as cancer, infection, sepsis, and so on.^14^ In solid tumors, MDSCs accumulation is associated with advanced disease stage and poor patient.^28^ According to retrospective studies, decreased HLA‐DR expression on monocytes represents monocyte anergy and is a reliable predictor of sepsis‐induced immunosuppression.^29^ Furthermore, delayed persistence of elevated M‐MDSCs is associated with negative outcomes in septic shock.^30^ In autoimmune diseases, a similar pattern of results was obtained. The clinical course of experimental autoimmune encephalomyelitis (EAE) appeared to be less severe as MDSCs became more abundant, along with milder histopathological affectation in the inflamed CNS.^31^ Peripheral MDSCs were found to be enriched in inflammatory bowel disease (IBD) patients during the effector phase of the disease.^32^ The findings indicate that MDSCs play a role in the progression of EAM. The positive relationship between splenic MDSCs and EAM clinical scores suggests that MDSCs could be used to assess EAM severity and disease prognosis. Additionally, LVEF/LVFS and LVAW/LVID were measured as the systolic function and ventricular remodeling in myocarditis progression. Our findings showed that LVEF, LVFS, and cardiac output declined significantly together with the incrassation of LVAW. Consistent with the percentage of MDSCs in heart, MDSCs seem to be a potential indicator for determining the risk of impairing systolic function. MDSCs' primary function in immune responses is to suppress effector T and B cells while inducing Treg differentiation to prevent excessive inflammation.^33^, ^34^ Th17 cells, as a pro‐inflammatory subset, protect against pathogens and participate in immune responses, whereas Tregs regulate the expansion and activation of autoreactive effector T cells to maintain immune tolerance.^5^, ^35^ The balance of Th17 and Treg cells is widely acknowledged to be closely related to disease activity, which has an impact on the onset of autoimmune and inflammatory diseases.^5^, ^36^ The ratio of Th17/Treg cells in the periphery was increased in pediatric psoriasis patients and was positively correlated with disease severity.^37^ The ratio of Th17/Treg cells was elevated in Graves' disease patients with higher clinical activity scores compared with those in healthy Controls.^38^ As previously stated, we hypothesized that MDSCs might play an important role in the development of EAM by mediating the balance of Th17 cells and Tregs.

To eliminate experimental setting differences, we used two widely used methods to detect MDSCs regulation on immune responses and EAM progression. Our study found that simply AT or depletion of MDSCs could correct Th17 cell/Treg imbalance and ameliorate the severity of myocarditis in EAM mice. Another novel finding was that AT of MDSCs followed by depletion triggered the imbalance of Th17 cells and Tregs, contributing to a degree of inflammation in EAM. Surprisingly, the ratio of Th17/Treg cells correlates with the severity of myocarditis. These findings debunk the widely held belief that MDSCs suppress immune responses. The tendency of pro‐inflammatory IL‐1β and anti‐inflammatory IL‐10 in early EAM stages of MDSCs transfer was opposed to the later EAM stages of MDSCs transfer. These results also suggested that MDSCs played a vital role in cytokine profiles to control inflammation state and modulate immune response, thus maintaining a mild inflammation in EAM progress. Furthermore, there are only a few findings that support our conclusion. MDSCs could promote T‐cell apoptosis in the murine model of EAE, resulting in inflammation reduction, whereas a case can be made for MDSCs enhancing Th17 differentiation to accelerate disease progression.^16^, ^39^ Similar findings have been reported in murine model of collagen‐induced arthritis and lupus.^15^, ^23^, ^26^ MDSCs retain the ability to inhibit T‐cell proliferation in lupus‐prone mice at early stages, but this ability is replaced later by the regulatory balance of Th17 and Treg.^26^ MDSCs play two roles in autoimmune diseases.

Additionally, we conducted coculture experiments to observe the function of MDSCs regulating Th17 and Treg cells in vitro. The suppressive function of MDSCs on T‐cell proliferation reached a peak in parallel with the increase of MDSCs proportion at an early stage, whereas its suppressive function was destroyer impaired as MDSCs increased further. Similarly, in IBD patients, bone marrow derived‐MDSCs switched phenotype and lost their suppressive properties due to a lack of CEB expression in a colonic inflammatory milieu.^32^ In secondary progressive multiple sclerosis, M‐MDSCs promoted T‐cell proliferation via transforming into a pro‐inflammatory phenotype with decreased CD163 surface expression and reduced mRNA expression of IL‐10 and HMOX‐1.^40^ A popular explanation is that MDSCs could modulate the proliferation of T cells through several different mechanisms according to the environmental milieu and disease pathology.^41^ The function of MDSCs on T‐cell expansion in autoimmune disease and inflammation order is dictated by distinct inflammatory signals and is reversible depending on the environmental milieu.^42^, ^43^ Given the expansion of CD4^+^ T cells in the Th17‐ or Treg‐polarizing conditions, the impaired suppressive activity is due to the local microenvironment to some extent.

Furthermore, under Th17‐polarizing conditions, MDSCs exhibited a pro‐inflammatory feature of promoting Th17 cell differentiation, whereas Tregs' inductive capacity was abolished. This is consistent with MDSCs inducing Th17 responses in systemic lupus erythematosus have an Ag‐1‐dependent effect.^44^ In patients with primary membranous nephropathy, increased expression of IL‐6 secreted by MDSCs expands MDSCs and increases Arg‐1 production for Th17 cell differentiation and IL‐17A production.^45^ PMN‐MDSCs inhibit TGF‐1‐induced differentiation of naïve T cells into Foxp3‐expressing iTregs in tumor‐bearing mice via ROS and IDO, and this occurs early in the differentiation process.^18^ Based on these findings, the mixed phenotype and cytokine expression of MDSCs could explain their inability to maintain immunosuppression.^26^, ^40^, ^46^, ^47^

The function of MDSCs in autoimmune diseases is not limited by their diversity. In sepsis and trauma, persistent inflammation causes MDSCs switching from a pro‐inflammatory cytokine profile, characterized by early TNF‐α, IL‐6, and IL‐12 secretion to a late anti‐inflammatory cytokine profile characterized by TGF‐β and IL‐10.^48^, ^49^ MDSCs killed *Leishmania major* parasites via NO secretion during experimental leishmaniasis and improved parasite clearance in skin lesions, enhancing resistance to *L. major*.^12^ As a first barrier against microbial invasion, MDSCs could produce plenty of bactericidal molecules like reactive oxygen species (ROS) and RNS and alleviate excessive inflammation associated with early organ dysfunctions.^14^ In active tuberculosis, MDSCs could harbor *M. tuberculosis* as reservoirs for *M. tuberculosis* survival.^13^ As a result, the function of MDSCs in various diseases is diverse and plastic.

However, some limitations must be mentioned. First, our study was mainly focused on the influence of MDSCs on Th17/Treg balance. The mechanisms, including the confused phenotypic and cytokine expression of MDSCs, require further investigation. Second, PMN‐MDSCs and neutrophils have similar morphological and phenotypic features but different biological functions, making it to differentiate these cells based on the universal phenotype.^50^, ^51^ Some evidence suggests that PMN‐MDSCs are pathologically activated neutrophils that exert pro‐inflammatory effects on T cells. Third, myocarditis is a three‐stage process that includes viral infections, autoimmune reactions, and ventricular remodeling, whereas the EAM model only mimics the immunologic activation of the disease process, which includes autoreactive T cells, cytokine activation, and cross‐reacting antibodies.

Taken together, the findings show that MDSC expansion correlates with the severity of myocarditis in EAM mice, and that the role of MDSCs in EAM is more of an immune regulator than an immunosuppressor. MDSCs maintain a mild inflammation in vivo and in vitro by shifting the balance between Th17 cells and Tregs via suppressive and inductive capacity. MDSCs, in particular, retain pro‐inflammatory capacity, while suppressing excessive inflammation during the peak of myocarditis. The functional diversity of MDSCs may be influenced by the environment disease progression. Given the plastic function of MDSCs, the use of MDSCs as an immunotherapy for the reduction of inflammation in autoimmune diseases should be approached with caution.

## AUTHOR CONTRIBUTIONS

Xin Xiong and Yange Wang performed the research and analyzed the data. Mengjia Yu and Longxian Cheng designed the research study and produced the initial draft of the manuscript. Dinghang Wang wrote the paper. All authors have read and approved the final submitted manuscript.

## CONFLICT OF INTEREST STATEMENT

The authors declare no conflict of interest.

## Supporting information

Figure S1 Ultrasound data acquisition and analysis of control and EAM mice. (A) Short axis view for M‐mode for the measurement of LV dimensions and systolic performance. (B) Serial echocardiographic results. At least three independent experiments have been carried out. * *p* < 0.05, *ns* indicates not significant. EF, left ventricular ejection fraction; FS, left ventricular functional shorting; LVAW(d/s), left ventricular anterior wall, diastolic/systolic; LVID(d), left ventricular internal dimension, diastolic.

Figure S2 The pro‐inflammatory and anti‐inflammatory cytokines in spleens of each group (n≧4). (A) Expression of TNF‐α, IL‐1β, IL‐10 and TGF‐β genes after MDSCs transfer in the early stages of EAM; (B) Expression of TNF‐α, IL‐1β, IL‐10 and TGF‐β genes in after MDSCs transfer in the later stages of EAM. Values are means ± SEM. * *p* < 0.05, ** *p* < 0.01.

Figure S3 The expression of Foxp3 in spleens of each group (n≧3). Values are means ± SEM. * *p* < 0.05, *ns* indicates not significant.

Figure S4 MDSCs control T‐cell development in T‐cell proliferation assays. (A) Sorted MDSCs were co‐cultured for 72–96 hours in different conditions with CFSE‐labeled splenocytes from Control mice at the indicated ratios. (B) Flow cytometry was used to assess the ratios of Th17 and Treg cells in co‐culturing CD4^+^ T cells with MDSCs at a 4:1 ratio. (C) ELISA was used to test the supernatants from co‐cultures of CD4 + T cells and MDSCs at a 4:1 ratio for IFN‐γ, IL‐1β, IL‐6, IL‐10, and TGF‐β concentrations (n = 3‐6). At least three independent experiments have been carried out. * *p* < 0.05, ** *p* < 0.01, *ns* indicates not significant.

## Data Availability

The data that support the findings of this study are available from the corresponding author upon reasonable request.
