# Supplementary figures and images for: Th17/Treg balance is regulated by myeloid‐derived suppressor cells in experimental autoimmune myocarditis
Source: Immun Inflamm Dis. 2023 Jun 14;11(6):e872. doi: 10.1002/iid3.872 (PMC10266145; doi:10.1002/iid3.872)

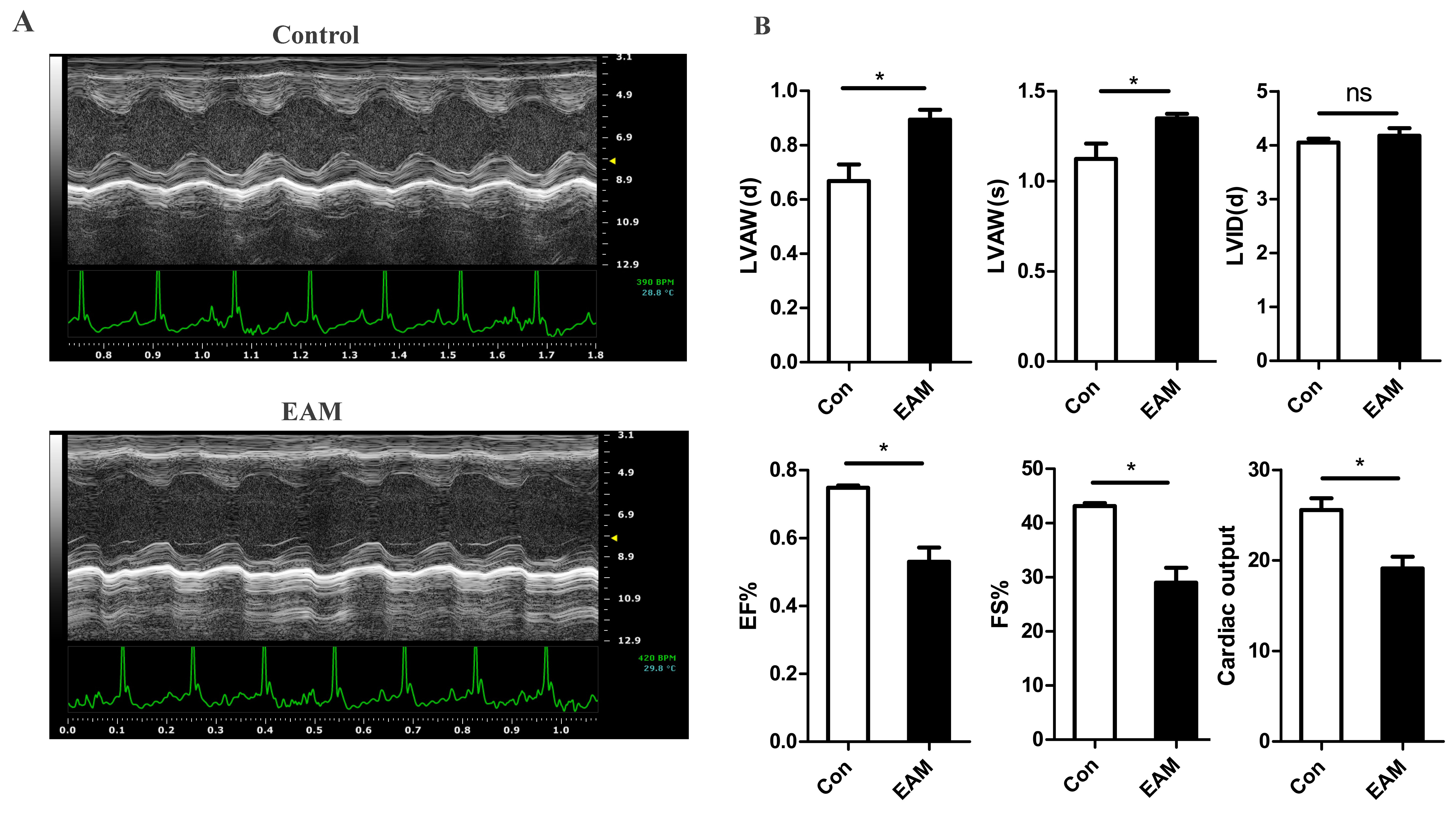

Supplement: Supplementary file 1 — Figure S1 Ultrasound data acquisition and analysis of control and EAM mice. (A) Short axis view for M‐mode for the measurement of LV dimensions and systolic performance. (B) Serial echocardiographic results. At least three independent experiments have been carried out. * p < 0.05, ns indicates not significant. EF, left ventricular ejection fraction; FS, left ventricular functional shorting; LVAW(d/s), left ventricular anterior wall, diastolic/systolic; LVID(d), left ventricular internal dimension, diastolic. [file IID3-11-e872-s002.jpg]

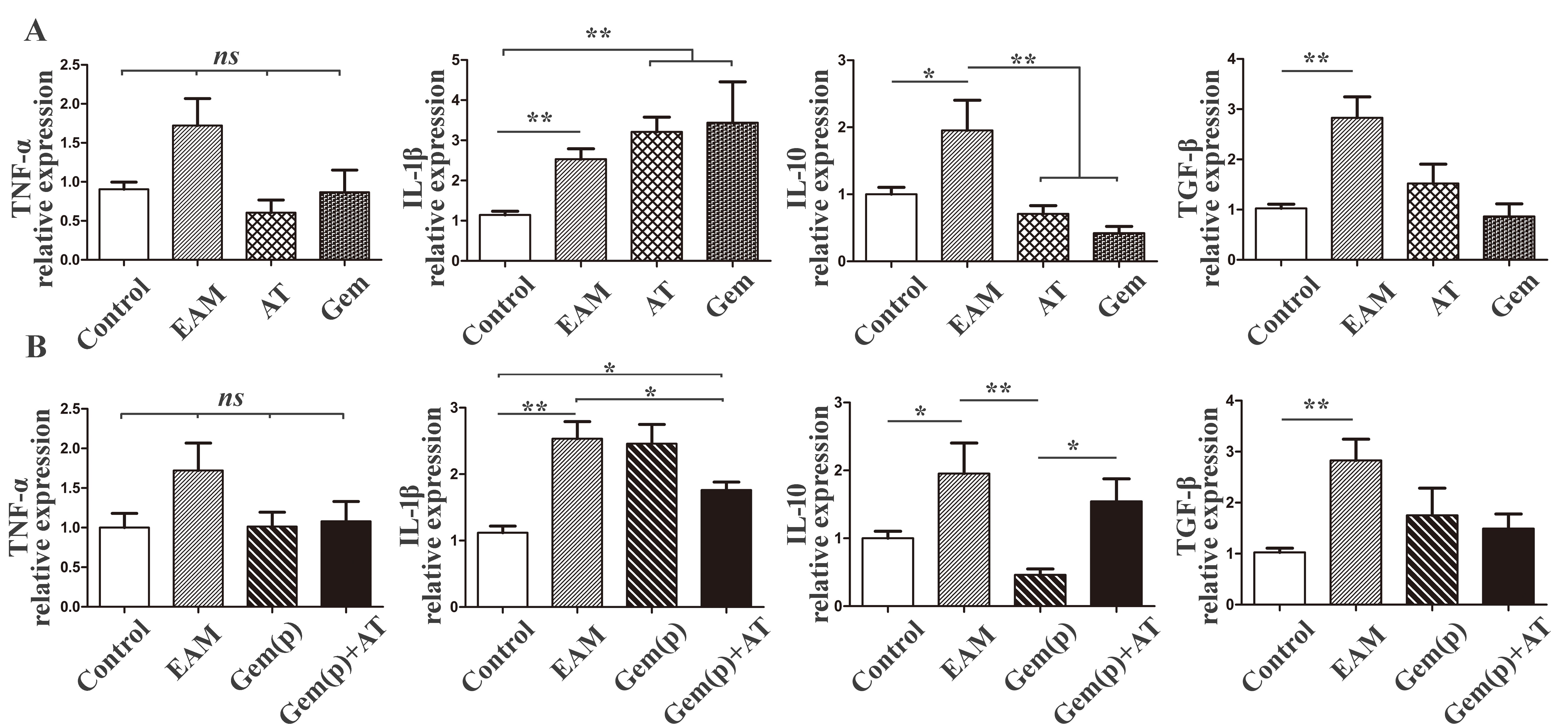

Supplement: Supplementary file 2 — Figure S2 The pro‐inflammatory and anti‐inflammatory cytokines in spleens of each group (n≧4). (A) Expression of TNF‐α, IL‐1β, IL‐10 and TGF‐β genes after MDSCs transfer in the early stages of EAM; (B) Expression of TNF‐α, IL‐1β, IL‐10 and TGF‐β genes in after MDSCs transfer in the later stages of EAM. Values are means ± SEM. * p < 0.05, ** p < 0.01. [file IID3-11-e872-s003.jpg]

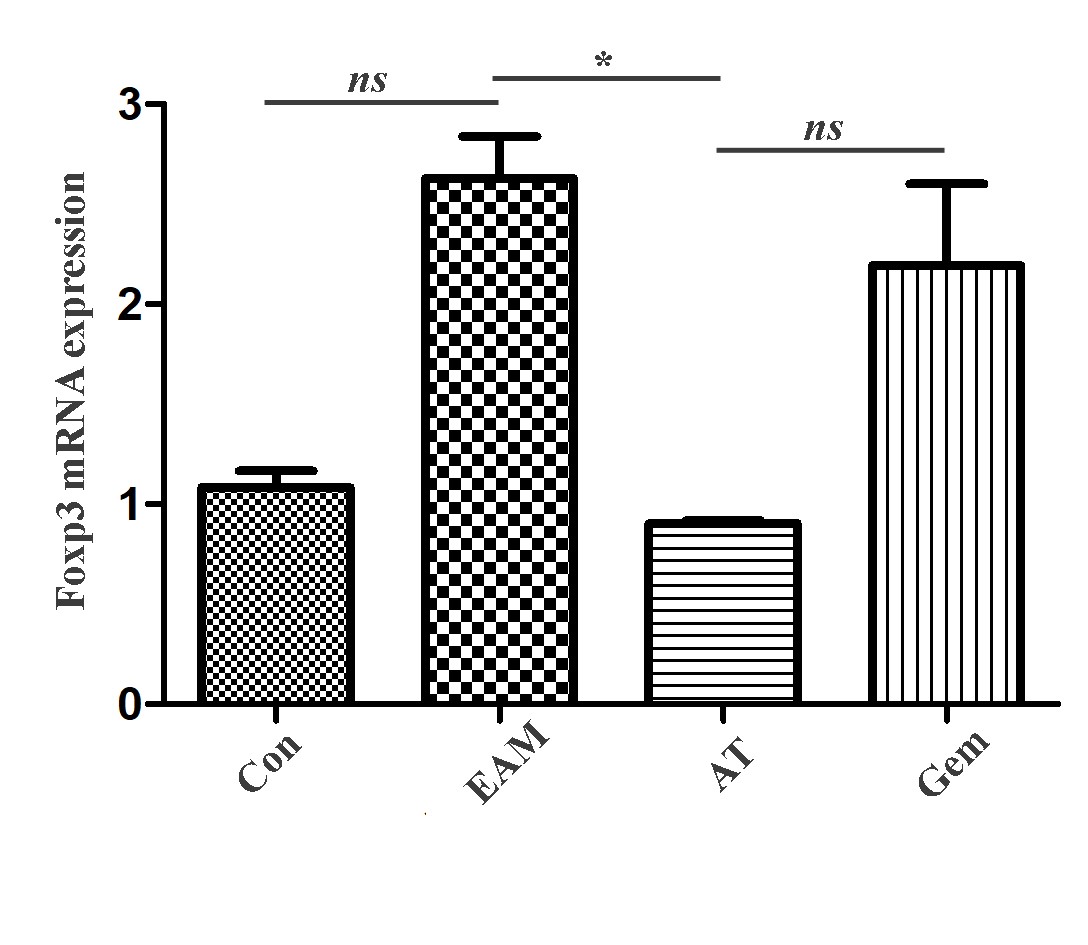

Supplement: Supplementary file 3 — Figure S3 The expression of Foxp3 in spleens of each group (n≧3). Values are means ± SEM. * p < 0.05, ns indicates not significant. [file IID3-11-e872-s001.jpg]

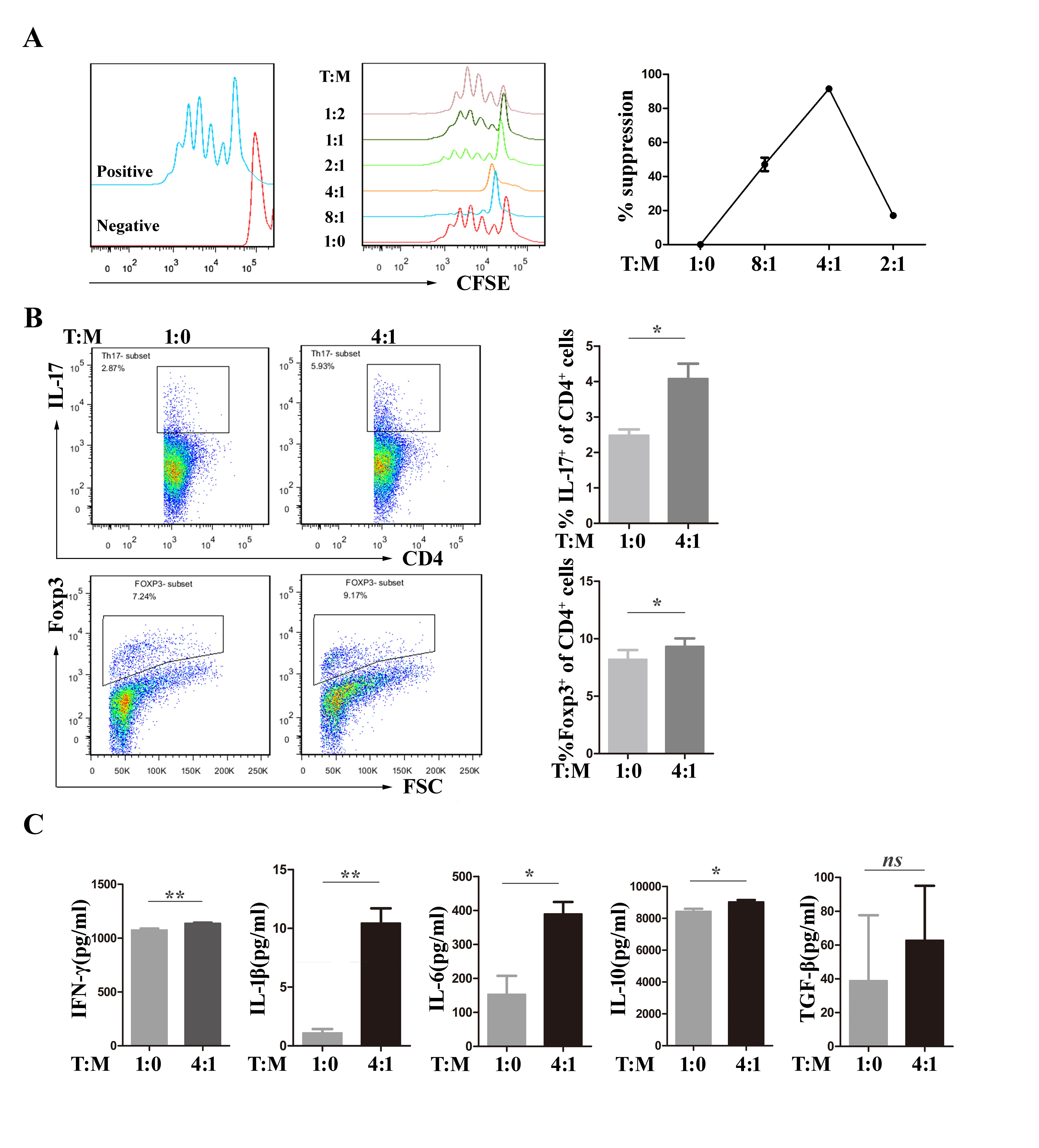

Supplement: Supplementary file 4 — Figure S4 MDSCs control T‐cell development in T‐cell proliferation assays. (A) Sorted MDSCs were co‐cultured for 72–96 hours in different conditions with CFSE‐labeled splenocytes from Control mice at the indicated ratios. (B) Flow cytometry was used to assess the ratios of Th17 and Treg cells in co‐culturing CD4+ T cells with MDSCs at a 4:1 ratio. (C) ELISA was used to test the supernatants from co‐cultures of CD4 + T cells and MDSCs at a 4:1 ratio for IFN‐γ, IL‐1β, IL‐6, IL‐10, and TGF‐β concentrations (n = 3‐6). At least three independent experiments have been carried out. * p < 0.05, ** p < 0.01, ns indicates not significant. [file IID3-11-e872-s004.jpg]
